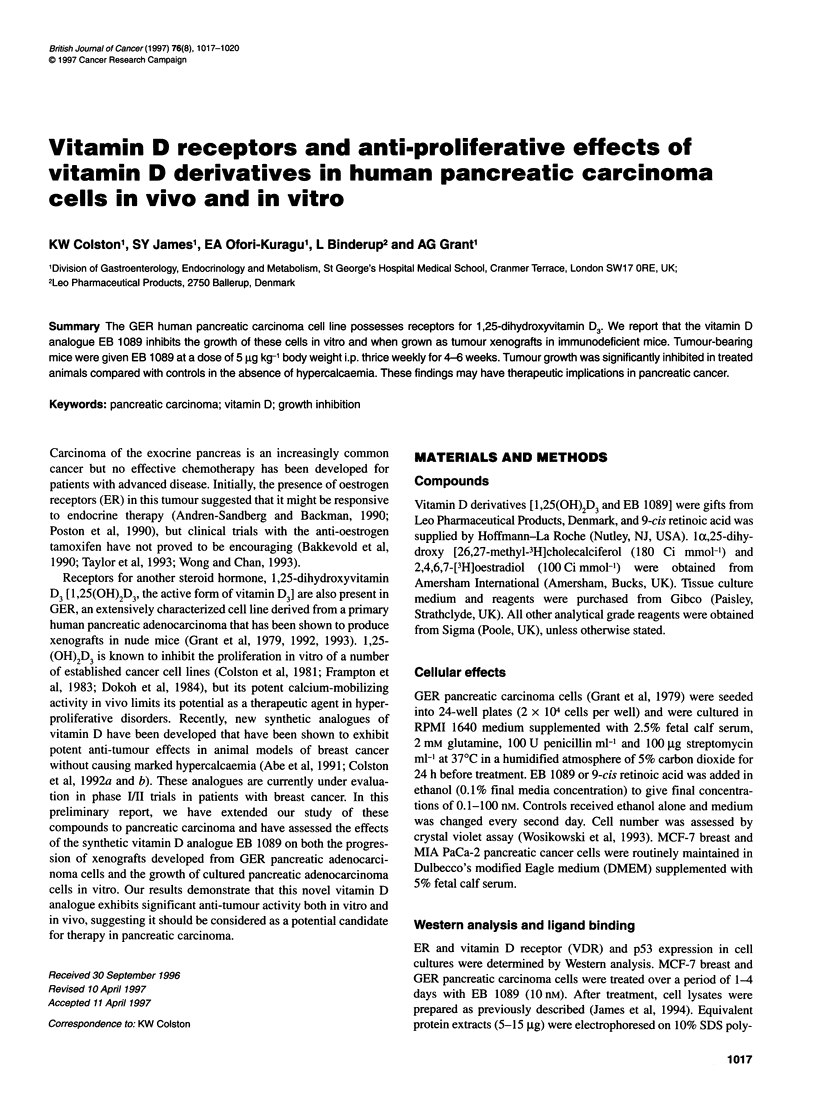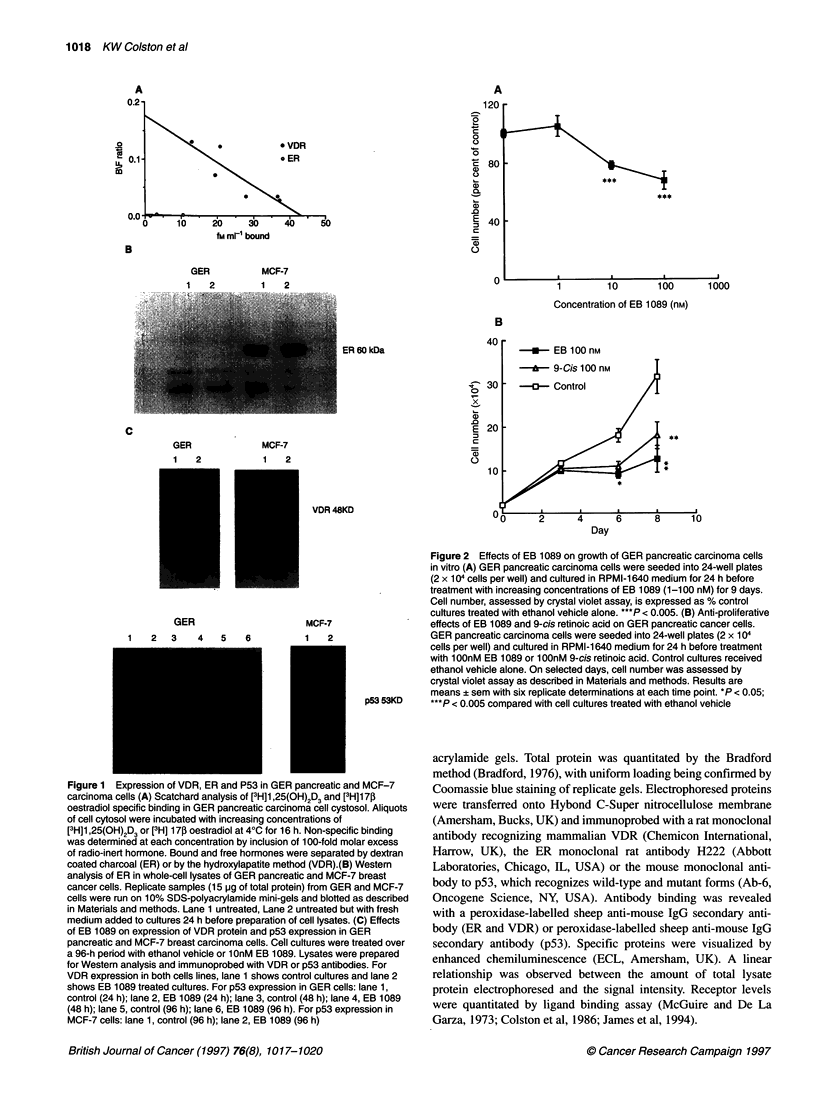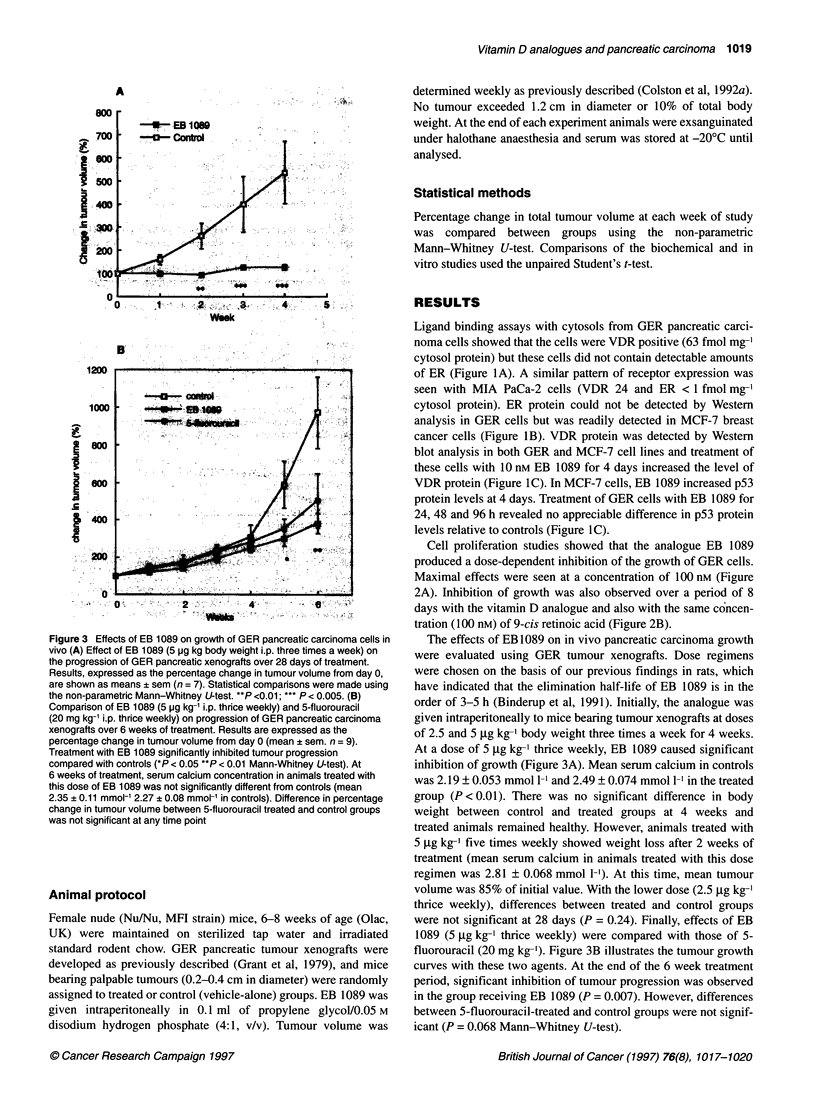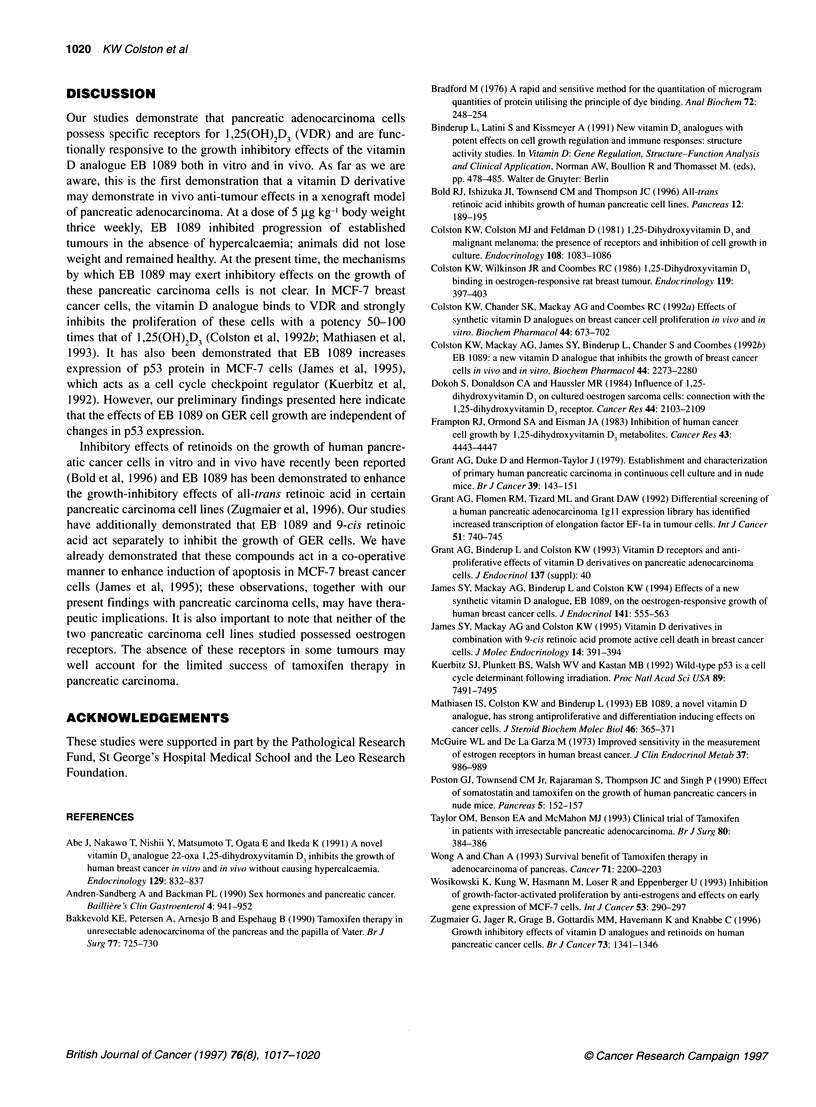# Vitamin D receptors and anti-proliferative effects of vitamin D derivatives in human pancreatic carcinoma cells in vivo and in vitro.

**DOI:** 10.1038/bjc.1997.501

**Published:** 1997

**Authors:** K. W. Colston, S. Y. James, E. A. Ofori-Kuragu, L. Binderup, A. G. Grant

**Affiliations:** Division of Gastroenterology, Endocrinology and Metabolism, St George's Hospital Medical School, London, UK.

## Abstract

**Images:**


British Journal of Cancer (1997) 76(8), 1017–1020
© 1997 Cancer Research Campaign

### Vitamin D receptors and anti-proliferative effects of vitamin D derivatives in human pancreatic carcinoma cells in vivo and in vitro

**KW Colston[1], SY James[1], EA Ofori-Kuragu[1], L Binderup[2] and AG Grant[1]**

[1]Division of Gastroenterology, Endocrinology and Metabolism, St George's Hospital Medical School, Cranmer Terrace, London SW17 0RE, UK; [2]Leo Pharmaceutical Products, 2750 Ballerup, Denmark

**Summary** The GER human pancreatic carcinoma cell line possesses receptors for 1,25-dihydroxyvitamin $D_3$. We report that the vitamin D analogue EB 1089 inhibits the growth of these cells in vitro and when grown as tumour xenografts in immunodeficient mice. Tumour-bearing mice were given EB 1089 at a dose of 5 μg $kg^{-1}$ body weight i.p. thrice weekly for 4–6 weeks. Tumour growth was significantly inhibited in treated animals compared with controls in the absence of hypercalcaemia. These findings may have therapeutic implications in pancreatic cancer.

**Keywords:** pancreatic carcinoma; vitamin D; growth inhibition

Carcinoma of the exocrine pancreas is an increasingly common cancer but no effective chemotherapy has been developed for patients with advanced disease. Initially, the presence of oestrogen receptors (ER) in this tumour suggested that it might be responsive to endocrine therapy (Andren-Sandberg and Backman, 1990; Poston et al, 1990), but clinical trials with the anti-oestrogen tamoxifen have not proved to be encouraging (Bakkevold et al, 1990; Taylor et al, 1993; Wong and Chan, 1993).

Receptors for another steroid hormone, 1,25-dihydroxyvitamin $D_3$ [$1,25(OH)_2D_3$, the active form of vitamin $D_3$] are also present in GER, an extensively characterized cell line derived from a primary human pancreatic adenocarcinoma that has been shown to produce xenografts in nude mice (Grant et al, 1979, 1992, 1993). $1,25(OH)_2D_3$ is known to inhibit the proliferation in vitro of a number of established cancer cell lines (Colston et al, 1981; Frampton et al, 1983; Dokoh et al, 1984), but its potent calcium-mobilizing activity in vivo limits its potential as a therapeutic agent in hyperproliferative disorders. Recently, new synthetic analogues of vitamin D have been developed that have been shown to exhibit potent anti-tumour effects in animal models of breast cancer without causing marked hypercalcaemia (Abe et al, 1991; Colston et al, 1992*a* and *b*). These analogues are currently under evaluation in phase I/II trials in patients with breast cancer. In this preliminary report, we have extended our study of these compounds to pancreatic carcinoma and have assessed the effects of the synthetic vitamin D analogue EB 1089 on both the progression of xenografts developed from GER pancreatic adenocarcinoma cells and the growth of cultured pancreatic adenocarcinoma cells in vitro. Our results demonstrate that this novel vitamin D analogue exhibits significant anti-tumour activity both in vitro and in vivo, suggesting it should be considered as a potential candidate for therapy in pancreatic carcinoma.

*Received 30 September 1996*
*Revised 10 April 1997*
*Accepted 11 April 1997*

*Correspondence to:* KW Colston

### MATERIALS AND METHODS

### Compounds

Vitamin D derivatives [$1,25(OH)_2D_3$ and EB 1089] were gifts from Leo Pharmaceutical Products, Denmark, and 9-*cis* retinoic acid was supplied by Hoffmann–La Roche (Nutley, NJ, USA). 1α,25-dihydroxy [26,27-methyl-$^3$H]cholecalciferol (180 Ci $mmol^{-1}$) and 2,4,6,7-[$^3$H]oestradiol (100 Ci $mmol^{-1}$) were obtained from Amersham International (Amersham, Bucks, UK). Tissue culture medium and reagents were purchased from Gibco (Paisley, Strathclyde, UK). All other analytical grade reagents were obtained from Sigma (Poole, UK), unless otherwise stated.

### Cellular effects

GER pancreatic carcinoma cells (Grant et al, 1979) were seeded into 24-well plates ($2 \times 10^4$ cells per well) and were cultured in RPMI 1640 medium supplemented with 2.5% fetal calf serum, 2 mM glutamine, 100 U penicillin $ml^{-1}$ and 100 μg streptomycin $ml^{-1}$ at 37°C in a humidified atmosphere of 5% carbon dioxide for 24 h before treatment. EB 1089 or 9-*cis* retinoic acid was added in ethanol (0.1% final media concentration) to give final concentrations of 0.1–100 nM. Controls received ethanol alone and medium was changed every second day. Cell number was assessed by crystal violet assay (Wosikowski et al, 1993). MCF-7 breast and MIA PaCa-2 pancreatic cancer cells were routinely maintained in Dulbecco's modified Eagle medium (DMEM) supplemented with 5% fetal calf serum.

### Western analysis and ligand binding

ER and vitamin D receptor (VDR) and p53 expression in cell cultures were determined by Western analysis. MCF-7 breast and GER pancreatic carcinoma cells were treated over a period of 1–4 days with EB 1089 (10 nM). After treatment, cell lysates were prepared as previously described (James et al, 1994). Equivalent protein extracts (5–15 μg) were electrophoresed on 10% SDS poly-

**Figure 1** Expression of VDR, ER and P53 in GER pancreatic and MCF-7 carcinoma cells (**A**) Scatchard analysis of [$^3$H]1,25(OH)$_2$D$_3$ and [$^3$H]17β oestradiol specific binding in GER pancreatic carcinoma cell cystosol. Aliquots of cell cytosol were incubated with increasing concentrations of [$^3$H]1,25(OH)$_2$D$_3$ or [$^3$H] 17β oestradiol at 4°C for 16 h. Non-specific binding was determined at each concentration by inclusion of 100-fold molar excess of radio-inert hormone. Bound and free hormones were separated by dextran coated charcoal (ER) or by the hydroxylapatite method (VDR).(**B**) Western analysis of ER in whole-cell lysates of GER pancreatic and MCF-7 breast cancer cells. Replicate samples (15 µg of total protein) from GER and MCF-7 cells were run on 10% SDS-polyacrylamide mini-gels and blotted as described in Materials and methods. Lane 1 untreated, Lane 2 untreated but with fresh medium added to cultures 24 h before preparation of cell lysates. (**C**) Effects of EB 1089 on expression of VDR protein and p53 expression in GER pancreatic and MCF-7 breast carcinoma cells. Cell cultures were treated over a 96-h period with ethanol vehicle or 10nM EB 1089. Lysates were prepared for Western analysis and immunoprobed with VDR or p53 antibodies. For VDR expression in both cells lines, lane 1 shows control cultures and lane 2 shows EB 1089 treated cultures. For p53 expression in GER cells: lane 1, control (24 h); lane 2, EB 1089 (24 h); lane 3, control (48 h); lane 4, EB 1089 (48 h); lane 5, control (96 h); lane 6, EB 1089 (96 h). For p53 expression in MCF-7 cells: lane 1, control (96 h); lane 2, EB 1089 (96 h)

**Figure 2** Effects of EB 1089 on growth of GER pancreatic carcinoma cells in vitro (**A**) GER pancreatic carcinoma cells were seeded into 24-well plates ($2 \times 10^4$ cells per well) and cultured in RPMI-1640 medium for 24 h before treatment with increasing concentrations of EB 1089 (1–100 nM) for 9 days. Cell number, assessed by crystal violet assay, is expressed as % control cultures treated with ethanol vehicle alone. ***$P < 0.005$. (**B**) Anti-proliferative effects of EB 1089 and 9-*cis* retinoic acid on GER pancreatic cancer cells. GER pancreatic carcinoma cells were seeded into 24-well plates ($2 \times 10^4$ cells per well) and cultured in RPMI-1640 medium for 24 h before treatment with 100nM EB 1089 or 100nM 9-*cis* retinoic acid. Control cultures received ethanol vehicle alone. On selected days, cell number was assessed by crystal violet assay as described in Materials and methods. Results are means ± sem with six replicate determinations at each time point. *$P < 0.05$; ***$P < 0.005$ compared with cell cultures treated with ethanol vehicle acrylamide gels. Total protein was quantitated by the Bradford method (Bradford, 1976), with uniform loading being confirmed by Coomassie blue staining of replicate gels. Electrophoresed proteins were transferred onto Hybond C-Super nitrocellulose membrane (Amersham, Bucks, UK) and immunoprobed with a rat monoclonal antibody recognizing mammalian VDR (Chemicon International, Harrow, UK), the ER monoclonal rat antibody H222 (Abbott Laboratories, Chicago, IL, USA) or the mouse monoclonal antibody to p53, which recognizes wild-type and mutant forms (Ab-6, Oncogene Science, NY, USA). Antibody binding was revealed with a peroxidase-labelled sheep anti-mouse IgG secondary antibody (ER and VDR) or peroxidase-labelled sheep anti-mouse IgG secondary antibody (p53). Specific proteins were visualized by enhanced chemiluminescence (ECL, Amersham, UK). A linear relationship was observed between the amount of total lysate protein electrophoresed and the signal intensity. Receptor levels were quantitated by ligand binding assay (McGuire and De La Garza, 1973; Colston et al, 1986; James et al, 1994).

© Cancer Research Campaign 1997

**Figure 3** Effects of EB 1089 on growth of GER pancreatic carcinoma cells in vivo (**A**) Effect of EB 1089 (5 µg kg body weight i.p. three times a week) on the progression of GER pancreatic xenografts over 28 days of treatment. Results, expressed as the percentage change in tumour volume from day 0, are shown as means ± sem ($n$ = 7). Statistical comparisons were made using the non-parametric Mann–Whitney $U$-test. **$P$ <0.01; *** $P$ < 0.005. (**B**) Comparison of EB 1089 (5 µg kg$^{-1}$ i.p. thrice weekly) and 5-fluorouracil (20 mg kg$^{-1}$ i.p. thrice weekly) on progression of GER pancreatic carcinoma xenografts over 6 weeks of treatment. Results are expressed as the percentage change in tumour volume from day 0 (mean ± sem. $n$ = 9). Treatment with EB 1089 significantly inhibited tumour progression compared with controls (*$P$ < 0.05 **$P$ < 0.01 Mann-Whitney $U$-test). At 6 weeks of treatment, serum calcium concentration in animals treated with this dose of EB 1089 was not significantly different from controls (mean 2.35 ± 0.11 mmol$^{-1}$ 2.27 ± 0.08 mmol$^{-1}$ in controls). Difference in percentage change in tumour volume between 5-fluorouracil treated and control groups was not significant at any time point

### Animal protocol

Female nude (Nu/Nu, MFI strain) mice, 6–8 weeks of age (Olac, UK) were maintained on sterilized tap water and irradiated standard rodent chow. GER pancreatic tumour xenografts were developed as previously described (Grant et al, 1979), and mice bearing palpable tumours (0.2–0.4 cm in diameter) were randomly assigned to treated or control (vehicle-alone) groups. EB 1089 was given intraperitoneally in 0.1 ml of propylene glycol/0.05 M disodium hydrogen phosphate (4:1, v/v). Tumour volume was determined weekly as previously described (Colston et al, 1992*a*). No tumour exceeded 1.2 cm in diameter or 10% of total body weight. At the end of each experiment animals were exsanguinated under halothane anaesthesia and serum was stored at –20°C until analysed.

### Statistical methods

Percentage change in total tumour volume at each week of study was compared between groups using the non-parametric Mann–Whitney $U$-test. Comparisons of the biochemical and in vitro studies used the unpaired Student's $t$-test.

### RESULTS

Ligand binding assays with cytosols from GER pancreatic carcinoma cells showed that the cells were VDR positive (63 fmol mg$^{-1}$ cytosol protein) but these cells did not contain detectable amounts of ER (Figure 1A). A similar pattern of receptor expression was seen with MIA PaCa-2 cells (VDR 24 and ER < 1 fmol mg$^{-1}$ cytosol protein). ER protein could not be detected by Western analysis in GER cells but was readily detected in MCF-7 breast cancer cells (Figure 1B). VDR protein was detected by Western blot analysis in both GER and MCF-7 cell lines and treatment of these cells with 10 nM EB 1089 for 4 days increased the level of VDR protein (Figure 1C). In MCF-7 cells, EB 1089 increased p53 protein levels at 4 days. Treatment of GER cells with EB 1089 for 24, 48 and 96 h revealed no appreciable difference in p53 protein levels relative to controls (Figure 1C).

Cell proliferation studies showed that the analogue EB 1089 produced a dose-dependent inhibition of the growth of GER cells. Maximal effects were seen at a concentration of 100 nM (Figure 2A). Inhibition of growth was also observed over a period of 8 days with the vitamin D analogue and also with the same concentration (100 nM) of 9-*cis* retinoic acid (Figure 2B).

The effects of EB1089 on in vivo pancreatic carcinoma growth were evaluated using GER tumour xenografts. Dose regimens were chosen on the basis of our previous findings in rats, which have indicated that the elimination half-life of EB 1089 is in the order of 3–5 h (Binderup et al, 1991). Initially, the analogue was given intraperitoneally to mice bearing tumour xenografts at doses of 2.5 and 5 µg kg$^{-1}$ body weight three times a week for 4 weeks. At a dose of 5 µg kg$^{-1}$ thrice weekly, EB 1089 caused significant inhibition of growth (Figure 3A). Mean serum calcium in controls was 2.19 ± 0.053 mmol l$^{-1}$ and 2.49 ± 0.074 mmol l$^{-1}$ in the treated group ($P$ < 0.01). There was no significant difference in body weight between control and treated groups at 4 weeks and treated animals remained healthy. However, animals treated with 5 µg kg$^{-1}$ five times weekly showed weight loss after 2 weeks of treatment (mean serum calcium in animals treated with this dose regimen was 2.81 ± 0.068 mmol l$^{-1}$). At this time, mean tumour volume was 85% of initial value. With the lower dose (2.5 µg kg$^{-1}$ thrice weekly), differences between treated and control groups were not significant at 28 days ($P$ = 0.24). Finally, effects of EB 1089 (5 µg kg$^{-1}$ thrice weekly) were compared with those of 5-fluorouracil (20 mg kg$^{-1}$). Figure 3B illustrates the tumour growth curves with these two agents. At the end of the 6 week treatment period, significant inhibition of tumour progression was observed in the group receiving EB 1089 ($P$ = 0.007). However, differences between 5-fluorouracil-treated and control groups were not significant ($P$ = 0.068 Mann–Whitney $U$-test).

© Cancer Research Campaign 1997

### DISCUSSION

Our studies demonstrate that pancreatic adenocarcinoma cells possess specific receptors for $1,25(OH)_2D_3$ (VDR) and are functionally responsive to the growth inhibitory effects of the vitamin D analogue EB 1089 both in vitro and in vivo. As far as we are aware, this is the first demonstration that a vitamin D derivative may demonstrate in vivo anti-tumour effects in a xenograft model of pancreatic adenocarcinoma. At a dose of 5 $\mu g\ kg^{-1}$ body weight thrice weekly, EB 1089 inhibited progression of established tumours in the absence of hypercalcaemia; animals did not lose weight and remained healthy. At the present time, the mechanisms by which EB 1089 may exert inhibitory effects on the growth of these pancreatic carcinoma cells is not clear. In MCF-7 breast cancer cells, the vitamin D analogue binds to VDR and strongly inhibits the proliferation of these cells with a potency 50–100 times that of $1,25(OH)_2D_3$ (Colston et al, 1992*b*; Mathiasen et al, 1993). It has also been demonstrated that EB 1089 increases expression of p53 protein in MCF-7 cells (James et al, 1995), which acts as a cell cycle checkpoint regulator (Kuerbitz et al, 1992). However, our preliminary findings presented here indicate that the effects of EB 1089 on GER cell growth are independent of changes in p53 expression.

Inhibitory effects of retinoids on the growth of human pancreatic cancer cells in vitro and in vivo have recently been reported (Bold et al, 1996) and EB 1089 has been demonstrated to enhance the growth-inhibitory effects of all-*trans* retinoic acid in certain pancreatic carcinoma cell lines (Zugmaier et al, 1996). Our studies have additionally demonstrated that EB 1089 and 9-*cis* retinoic acid act separately to inhibit the growth of GER cells. We have already demonstrated that these compounds act in a co-operative manner to enhance induction of apoptosis in MCF-7 breast cancer cells (James et al, 1995); these observations, together with our present findings with pancreatic carcinoma cells, may have therapeutic implications. It is also important to note that neither of the two pancreatic carcinoma cell lines studied possessed oestrogen receptors. The absence of these receptors in some tumours may well account for the limited success of tamoxifen therapy in pancreatic carcinoma.

### ACKNOWLEDGEMENTS

These studies were supported in part by the Pathological Research Fund, St George's Hospital Medical School and the Leo Research Foundation.

### REFERENCES

Abe J, Nakawo T, Nishii Y, Matsumoto T, Ogata E and Ikeda K (1991) A novel vitamin $D_3$ analogue 22-oxa 1,25-dihydroxyvitamin $D_3$ inhibits the growth of human breast cancer *in vitro* and *in vivo* without causing hypercalcaemia. *Endocrinology* **129**: 832–837

Andren-Sandberg A and Backman PL (1990) Sex hormones and pancreatic cancer. *Baillière's Clin Gastroenterol* **4**: 941–952

Bakkevold KE, Petersen A, Arnesjo B and Espehaug B (1990) Tamoxifen therapy in unresectable adenocarcinoma of the pancreas and the papilla of Vater. *Br J Surg* **77**: 725–730

Bradford M (1976) A rapid and sensitive method for the quantitation of microgram quantities of protein utilising the principle of dye binding. *Anal Biochem* **72**: 248–254

Binderup L, Latini S and Kissmeyer A (1991) New vitamin $D_3$ analogues with potent effects on cell growth regulation and immune responses: structure activity studies. In *Vitamin D: Gene Regulation, Structure–Function Analysis and Clinical Application*, Norman AW, Boullion R and Thomasset M. (eds), pp. 478–485. Walter de Gruyter: Berlin

Bold RJ, Ishizuka JI, Townsend CM and Thompson JC (1996) All-*trans* retinoic acid inhibits growth of human pancreatic cell lines. *Pancreas* **12**: 189–195

Colston KW, Colston MJ and Feldman D (1981) 1,25-Dihydroxyvitamin $D_3$ and malignant melanoma: the presence of receptors and inhibition of cell growth in culture. *Endocrinology* **108**: 1083–1086

Colston KW, Wilkinson JR and Coombes RC (1986) 1,25-Dihydroxyvitamin $D_3$ binding in oestrogen-responsive rat breast tumour. *Endocrinology* **119**: 397–403

Colston KW, Chander SK, Mackay AG and Coombes RC (1992*a*) Effects of synthetic vitamin D analogues on breast cancer cell proliferation *in vivo* and *in vitro*. *Biochem Pharmacol* **44**: 673–702

Colston KW, Mackay AG, James SY, Binderup L, Chander S and Coombes (1992*b*) EB 1089: a new vitamin D analogue that inhibits the growth of breast cancer cells *in vivo* and *in vitro*. *Biochem Pharmacol* **44**: 2273–2280

Dokoh S, Donaldson CA and Haussler MR (1984) Influence of 1,25-dihydroxyvitamin $D_3$ on cultured oestrogen sarcoma cells: connection with the 1,25-dihydroxyvitamin $D_3$ receptor. *Cancer Res* **44**: 2103–2109

Frampton RJ, Ormond SA and Eisman JA (1983) Inhibition of human cancer cell growth by 1,25-dihydroxyvitamin $D_3$ metabolites. *Cancer Res* **43**: 4443–4447

Grant AG, Duke D and Hermon-Taylor J (1979). Establishment and characterization of primary human pancreatic carcinoma in continuous cell culture and in nude mice. *Br J Cancer* **39**: 143–151

Grant AG, Flomen RM, Tizard ML and Grant DAW (1992) Differential screening of a human pancreatic adenocarcinoma 1g11 expression library has identified increased transcription of elongation factor EF-1a in tumour cells. *Int J Cancer* **51**: 740–745

Grant AG, Binderup L and Colston KW (1993) Vitamin D receptors and anti-proliferative effects of vitamin D derivatives on pancreatic adenocarcinoma cells. *J Endocrinol* **137** (suppl): 40

James SY, Mackay AG, Binderup L and Colston KW (1994) Effects of a new synthetic vitamin D analogue, EB 1089, on the oestrogen-responsive growth of human breast cancer cells. *J Endocrinol* **141**: 555–563

James SY, Mackay AG and Colston KW (1995) Vitamin D derivatives in combination with 9-*cis* retinoic acid promote active cell death in breast cancer cells. *J Molec Endocrinology* **14**: 391–394

Kuerbitz SJ, Plunkett BS, Walsh WV and Kastan MB (1992) Wild-type p53 is a cell cycle determinant following irradiation. *Proc Natl Acad Sci USA* **89**: 7491–7495

Mathiasen IS, Colston KW and Binderup L (1993) EB 1089, a novel vitamin D analogue, has strong antiproliferative and differentiation inducing effects on cancer cells. *J Steroid Biochem Molec Biol* **46**: 365–371

McGuire WL and De La Garza M (1973) Improved sensitivity in the measurement of estrogen receptors in human breast cancer. *J Clin Endocrinol Metab* **37**: 986–989

Poston GJ, Townsend CM Jr, Rajaraman S, Thompson JC and Singh P (1990) Effect of somatostatin and tamoxifen on the growth of human pancreatic cancers in nude mice. *Pancreas* **5**: 152–157

Taylor OM, Benson EA and McMahon MJ (1993) Clinical trial of Tamoxifen in patients with irresectable pancreatic adenocarcinoma. *Br J Surg* **80**: 384–386

Wong A and Chan A (1993) Survival benefit of Tamoxifen therapy in adenocarcinoma of pancreas. *Cancer* **71**: 2200–2203

Wosikowski K, Kung W, Hasmann M, Loser R and Eppenberger U (1993) Inhibition of growth-factor-activated proliferation by anti-estrogens and effects on early gene expression of MCF-7 cells. *Int J Cancer* **53**: 290–297

Zugmaier G, Jager R, Grage B, Gottardis MM, Havemann K and Knabbe C (1996) Growth inhibitory effects of vitamin D analogues and retinoids on human pancreatic cancer cells. *Br J Cancer* **73**: 1341–1346

© Cancer Research Campaign 1997